# Heteromeric Anopheline Odorant Receptors Exhibit Distinct Channel Properties

**DOI:** 10.1371/journal.pone.0028774

**Published:** 2011-12-09

**Authors:** Gregory M. Pask, Patrick L. Jones, Michael Rützler, David C. Rinker, Laurence J. Zwiebel

**Affiliations:** 1 Department of Biological Sciences, Vanderbilt University, Nashville, Tennessee, United States of America; 2 The Water and Salt Research Center, Institute of Anatomy, University of Aarhus, Aarhus C, Denmark; 3 Center for Human Genetics Research, Vanderbilt University Medical Center, Nashville, Tennessee, United States of America; 4 Department of Pharmacology, Centers for Molecular Neuroscience and Human Genetics Research, Institutes of Chemical Biology and Global Health and Program in Developmental Biology, Vanderbilt University Medical Center, Nashville, Tennessee, United States of America; University of Houston, United States of America

## Abstract

**Background:**

Insect odorant receptors (ORs) function as odorant-gated ion channels consisting of a conventional, odorant-binding OR and the Orco coreceptor. While Orco can function as a homomeric ion channel, the role(s) of the conventional OR in heteromeric OR complexes has largely focused only on odorant recognition.

**Results:**

To investigate other roles of odorant-binding ORs, we have employed patch clamp electrophysiology to investigate the properties of the channel pore of several OR complexes formed by a range of different odorant-specific *Anopheles gambiae* ORs (AgOrs) each paired with AgOrco. These studies reveal significant differences in cation permeability and ruthenium red susceptibility among different AgOr complexes.

**Conclusions:**

With observable differences in channel function, the data support a model in which the odorant-binding OR also affects the channel pore. The variable effect contributed by the conventional OR on the conductive properties of odorant-gated sensory channels adds additional complexity to insect olfactory signaling, with differences in odor coding beginning with ORs on the periphery of the olfactory system.

## Introduction

The ability to sense a wide range of distinct odorants relies on large families of cell surface odorant receptors (ORs) that are expressed on dendrites of olfactory receptor neurons (ORNs). In contrast to the GPCR-based ORs in vertebrates, insects have an alternative system of olfactory signal transduction that utilizes ligand-gated ion channels [1]–[3]. In addition, insects also utilize a class of variant ionotropic receptors (IRs) that act independently from ORs as chemosensory receptors [4]. Although the precise stoichiometry has not been established, functional insect OR complexes consist of a conventional OR, responsible for odorant recognition, and an extraordinarily conserved coreceptor OR, Orco. In *Drosophila*, Orco has been implicated in dendritic localization of the OR complex and its functional conservation has been demonstrated in *Orco* null mutant flies that have olfactory responses rescued by expression of Orco orthologs from other insects [5], [6]. Orco is critical for OR olfactory signaling, as conventional ORs are nonfunctional when expressed without Orco [5].

It has been demonstrated that Orco can also form functional homomeric channels when solely expressed in HEK cells [2], [3]. Additionally, a putative pore region in Orco has been identified on its similarity to a K^+^ channel selectivity filter [2]. However, when Orco is in complex with a conventional OR, the makeup of the ion channel pore remains unclear. Regarding Orco's contribution to the channel pore, only slight differences in cation permeability and channel blockade have been observed when varying Orco subunits have been paired with a conventional OR, most likely due to the high conservation across insect taxa [1], [7]. In the empty neuron system in *Drosophila*, the expression of different odorant-binding ORs imparts unique spontaneous ORN spike frequencies, suggesting that heteromeric OR complexes possess distinct conductive properties [8]. Within this context it is possible that Orco alone could form the ion channel pore, with the conventional OR providing distinct odorant recognition and channel gating domains. Conversely, both the Orco and conventional OR could form a single heteromultimeric structure that forms the channel pore and functions in odorant recognition/gating, comparable to the different subunits that comprise the pore of other, more characterized ligand-gated ion channels [9]–[11]. Additionally, certain subunits of cyclic-nucleotide gated (CNG) and transient receptor potential (TRP) channels can form functional homomeric channels, often with properties distinct from the heteromeric conformation [9], [10].

Olfactory signaling plays a critical role in mediating the vectorial capacity in the principal afrotropical malaria vector mosquito *Anopheles gambiae* [12]. By examining the potential for OR-specific properties of AgOr channel pores, these studies aim to develop a better understanding of the diverse molecular architecture of heteromeric OR complexes. Along with the ongoing efforts to characterize odorant sensitivity and tuning profiles in *An. gambiae* and other insects, these studies provide an enhanced understanding of the contribution of conventional ORs to channel function [8], [13], [14]. In light of our results, we propose a molecular model of insect OR function, where the odorant-binding OR also influences the conductive properties, and consequently the downstream odor coding capacity of odorant-evoked ORN signaling.

## Results

To determine the potential role of conventional OR subunits in forming the channel pore, we examined cation permeability and susceptibility to channel block across four conventional ORs from *An. gambiae,* each paired with AgOrco. The primary sequences and odorant sensitivities across these odorant-binding AgOrs are divergent, leading one to expect differences in conductive properties if the conventional AgOr contributes to the channel pore. In order to compare currents across different AgOr pairs that respond to different odorants, the recently identified Orco agonist, VUAA1, served as the control for potential agonist-related differences [3]. It is possible that AgOrco homomers may also exist in our cell lines expressing both AgOrco and another AgOr, which could potential affect interpretation of the VUAA1-based experiments. To address these concerns, each stable cell line uses the same insertion site and the identical dual promoter system. Importantly, AgOr complex properties were also assayed using odorants identified as strong agonists to assure that currents are not primarily due to homomeric AgOrco channels, which are non-responsive to the odorants used in this study (Figure S1).

The representative set of conventional AgOrs assayed in this study spans AgOrs 8, 10, 28, and 65, which are diverse in primary sequence (<20% identity), odorant-specificity, and expression [13]–[15]. In adult mosquitoes, AgOrs 8 and 28 are the only ORs expressed in the maxillary palp, while AgOrs 10 and 28 are both in the reduced set of ORs expressed during the larval stage [16], [17]. Furthermore, AgOr10 is one of the few ORs highly conserved across Anophelinae and Culicinae mosquitoes [18], [19]. From an odor-coding perspective, AgOr65 is narrowly tuned to eugenol, while AgOrs 10 and 28 respond to a wider variety of odorants [13], [14], [16].

**Figure 1:**
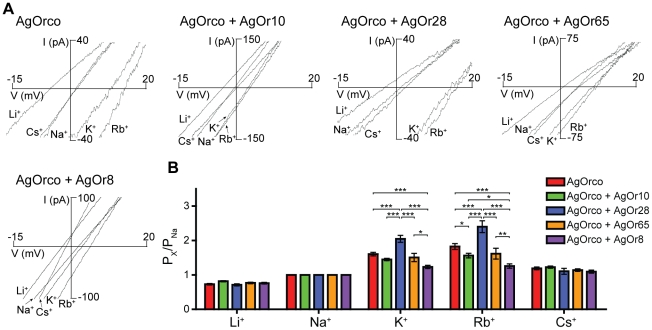
Monovalent cation permeation varies across AgOrs with VUAA1 agonism. (*A*) Representative VUAA1-induced currents across different AgOrs in extracellular solution containing 150 mM of the indicated monovalent cation and 100 µM VUAA1. (*B*) Histogram of the relative permeation of the monovalent cations to Na^+^ for each AgOr (n = 5 for each). Significance of the AgOr and the cation were determined by a two-factor ANOVA (p<0.0001 for both), and a Bonferroni correction was performed for individual comparisons (***  =  p<0.001, **  =  p<0.01, *  =  p<0.05).

The relative permeability of monovalent cations across different AgOr combinations functionally expressed in HEK cells was determined through whole-cell patch clamp electrophysiology. In these studies, agonist-induced currents were subjected to a voltage ramp to determine the reversal potential, where net current through the channel is zero, in the presence of a single monovalent cation. As seen in Figure 1A, the more permeable cations have rightward shifts in reversal potential. When the Orco agonist VUAA1 was applied, significant differences in the relative permeability of K^+^ and Rb^+^ were observed between different AgOrs paired with AgOrco, suggesting that VUAA1 is acting on heteromeric AgOR complexes, not simply AgOrco homomers (Figure 1B). For each AgOr combination, the same permeability sequence of Rb^+^≥K^+^ > Cs^+^ > Na^+^ > Li^+^ (Eisenman sequence III) was observed, which corresponds to a weak field strength binding site in the channel pore, where the permeability of the ion is largely determined by the hydration energy [20]–[22]. AgOrco + AgOr28-expressing cells were significantly more permeable to K^+^ and Rb^+^ with respective relative permeabilities to Na^+^ of 2.05±0.10 and 2.40±0.17.

**Figure 2 pone-0028774-g002:**
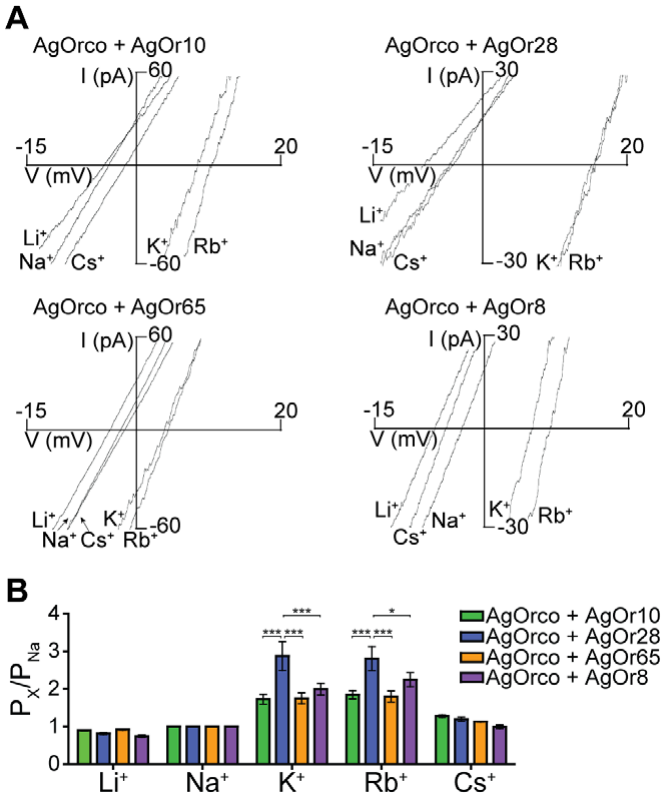
Odorant-induced monovalent permeation of heteromeric AgOrs. (*A*) Representative currents from AgOrs when activated by an odorant in extracellular solution containing 150 mM of the specified monovalent cation. AgOr:odorant pairs are as follows AgOr10:benzaldehyde (100 µM), AgOr28:2,4,5-trimethylthiazole (100 µM), AgOr65:eugenol (100 nM), and AgOr8:1-octen-3-ol (100 µM). (*B*) Histogram of the relative permeation of the monovalent cations to Na^+^ for each AgOr (n = 5 for each). Significance of the AgOr and the cation were determined by a two-factor ANOVA (p<0.0001 for both), and a Bonferroni correction was performed for individual comparisons (***  =  p<0.001, *  =  p<0.05).

When the same combinations of AgOrco + AgOr-expressing cells, excluding AgOrco alone, were assayed with strong odorant agonists specific to the conventional AgOr subunits, AgOrco + AgOr28 again displayed significantly higher permeabilities of K^+^ and Rb^+^, 2.87±0.38 and 2.80±0.32 (Figure 2). In some cases, agonist-specific differences in relative permeability were observed when comparing the odorant-induced currents to those from VUAA1 (Figure S2). These data suggest that channel gating mediated by either AgOrco or the conventional AgOr results in a different architecture of the channel pore, thus allowing particular ions to be more or less permeant.

**Figure 3 pone-0028774-g003:**
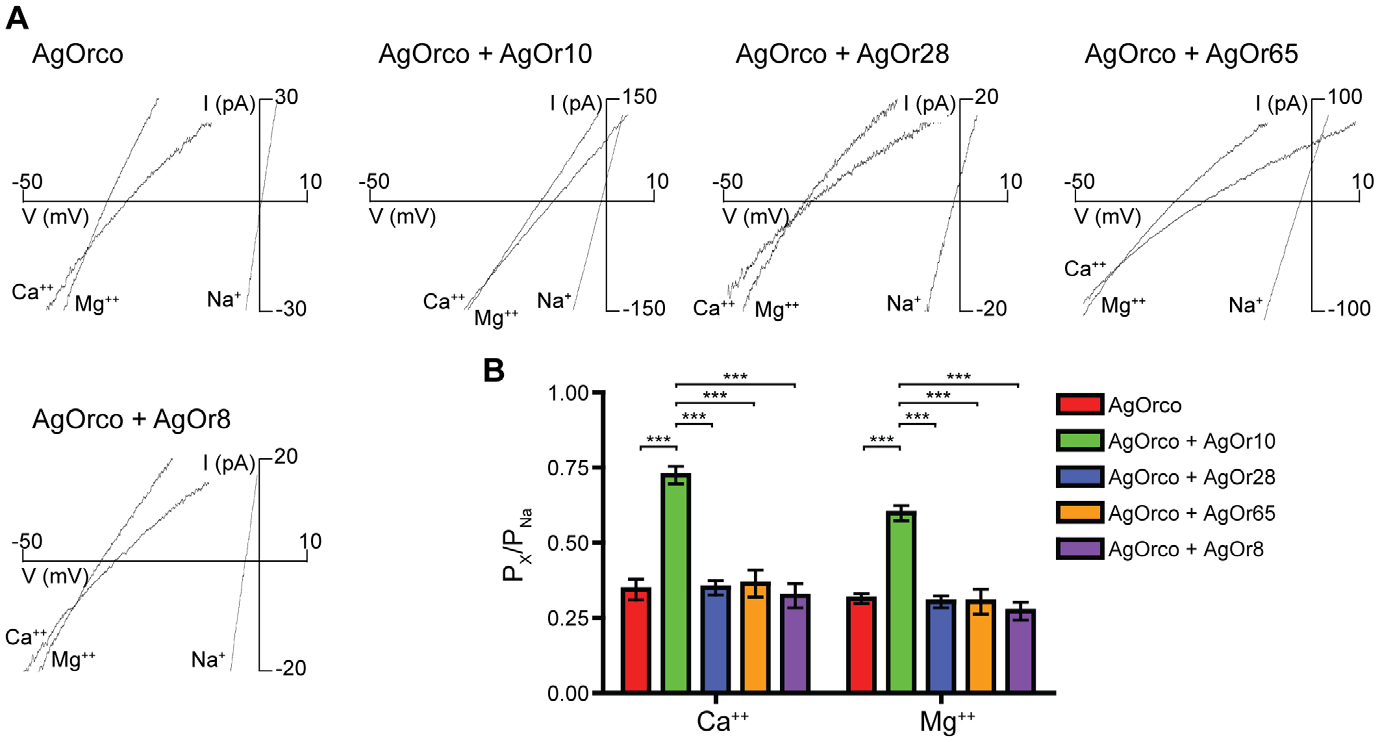
Divalent cation permeability between AgOrs activated by VUAA1. (*A*) Representative divalent cation currents from external solution containing 30 mM of either Ca^++^ or Mg^++^ and 100 µM VUAA1. Currents from 150 mM Na^+^ are included for comparison. (*B*) Histogram of the relative permeation of the divalent cations to Na^+^ for each AgOr (n = 5 for each). Significance of the AgOr and the cation were determined by a two-factor ANOVA (p<0.0001 for both), and a Bonferroni correction was performed for individual comparisons (***  =  p<0.001).

**Figure 4:**
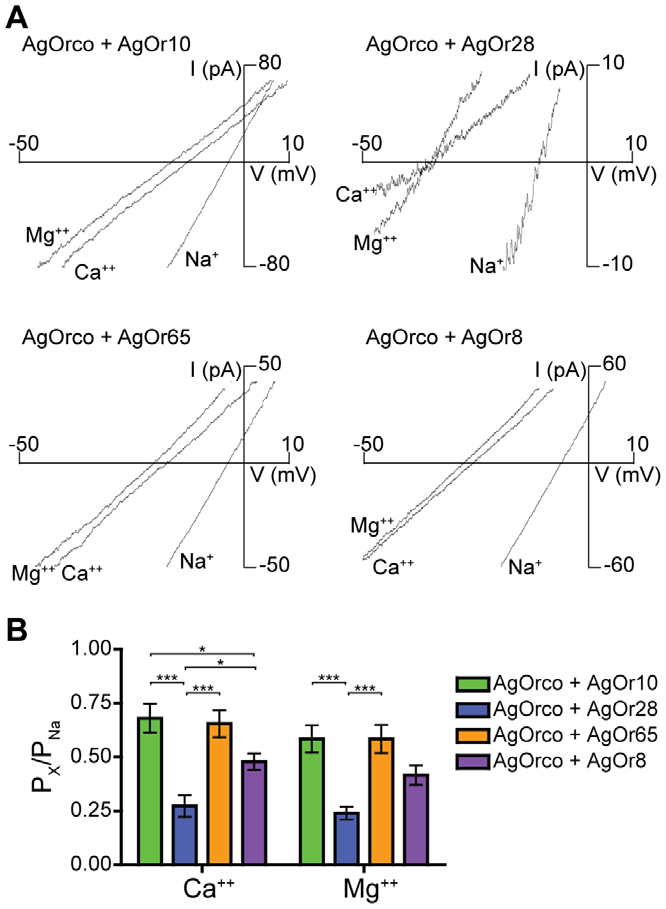
Divalent permeability differs between heteromeric AgOrs with odorant agonism. (*A*) Divalent currents from AgOrs in 30 mM Ca^++^ or Mg^++^ and the corresponding odorant. Currents from 150 mM Na^+^ are included for comparison. AgOr:odorant pairs are the same as in Figure 2. (*B*) Histogram of the relative permeation of the divalent cations to Na^+^ for each AgOr (n = 5 for each). Significance of the AgOr and the cation were determined by a two-factor ANOVA (p<0.0001 for both), and a Bonferroni correction was performed for individual comparisons (***  =  p<0.001, *  =  p<0.05).

Insect ORs are also permeable to divalent cations, previously demonstrated by Ca^++^ mobilization assays used to assess OR function [1]–[3], [19]. Extracellular solutions containing a single divalent cation were used to determine the relative permeability of Ca^++^ and Mg^++^ among the different AgOr cell lines as in Figures 1 and 2. In the context of VUAA1 agonism, both divalent cations were less permeable than Na^+^ across each AgOr combination (Figure 3). However, AgOrco + AgOr10 was significantly more permeable to both Ca^++^ and Mg^++^ than the other AgOrs with permeability ratios of 0.72±0.03 and 0.60±0.03, respectively. When activated by the odorant, Ca^++^ and Mg^++^ permeability was dependent on the conventional AgOr (Figure 4). In cells expressing AgOrco + AgOr65 and AgOrco + AgOr8, significant increases in permeability for both divalent cations were observed when compared to VUAA1 agonism, again demonstrating differences in permeability related to the agonist (Figure S3). Significant macroscopic currents were observed for all cations tested, confirming the role of insect ORs as non-selective cation channels, with a preference for monovalent over divalent cations.

Ruthenium red (RR) has been used as a blocker of insect ORs and other cation channels and is believed to bind to the extracellular entrance to the channel pore [1], [3], [7], [23], [24]. In addition to the differences in cation permeability, differences in the ability of RR to block VUAA1 or odorant-induced currents across different AgOr pairs would further support the hypothesis that the conventional odorant-binding ORs contribute to the OR ion channel pore.

**Figure 5:**
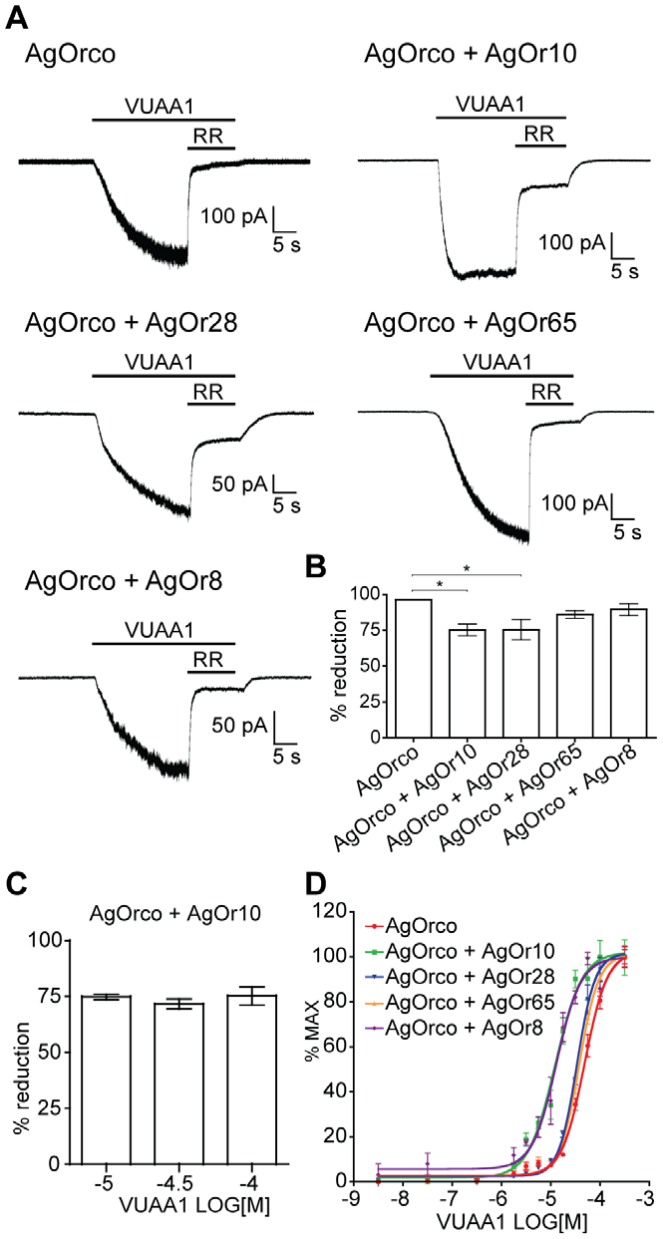
RR sensitivity varies across VUAA1-stimulated AgOrs. (*A*) Representative traces of macroscopic currents from 100 µM VUAA1, with subsequent current block by application of 100 µM RR. Holding potential for each recording is −60 mV. (*B*) The percent current reduction upon RR application across each AgOr combination (n = 5 for each). Statistical significance was determined by a one-factor ANOVA (p<0.01), and a Bonferroni correction was performed for individual comparisons (*  =  p<0.05). (*C*) RR (100 µM) sensitivity across varying concentrations of VUAA1 agonist in AgOrco + AgOr10 cells (n = 5). (*D*) Concentration-response curves generated from Ca^++^ imaging with AgOr cell lines in response to VUAA1 (n = 4). EC_50_ values for each AgOr complex: AgOrco, −4.31±0.03 logM; AgOrco + AgOr10, −4.91±0.05 logM; AgOrco + AgOr28, −4.47±0.02 logM; AgOrco + AgOr65, −4.42±0.02 logM; AgOrco + AgOr8, −4.88±0.05 logM. Statistical significance was determined by a one-factor ANOVA (p<0.0001), and individual comparisons (Bonferroni) resulted in two statistically different (p<0.001) groups *a* (AgOrco + AgOr10 and AgOrco + AgOr8) and *b* (AgOrco, AgOrco + AgOr28 and AgOrco + AgOr65).

In these studies, when VUAA1-currents were blocked by 100 µM RR, AgOrco + AgOr10 and AgOrco + AgOr28 were significantly less susceptible to RR blockade than AgOrco alone (Figure 5*A*–*B*). Furthermore, AgOrco + AgOr10 demonstrates significantly faster activation kinetics when compared to the other AgOrs, most likely due to the previously observed differences in sensitivity when compared to cells expressing AgOrco alone (Table S2) [3]. Varying the concentration of VUAA1 did not alter the sensitivity to RR, demonstrating that RR is noncompetitive with VUAA1 agonism (Figure 5*C*). In addition, each AgOr complex displayed concentration-dependent responses to VUAA1 in a Ca^++^-based imaging assay. Significantly different sensitivities to the Orco agonist were observed, further suggesting that different AgOrs for variant complexes.

**Figure 6:**
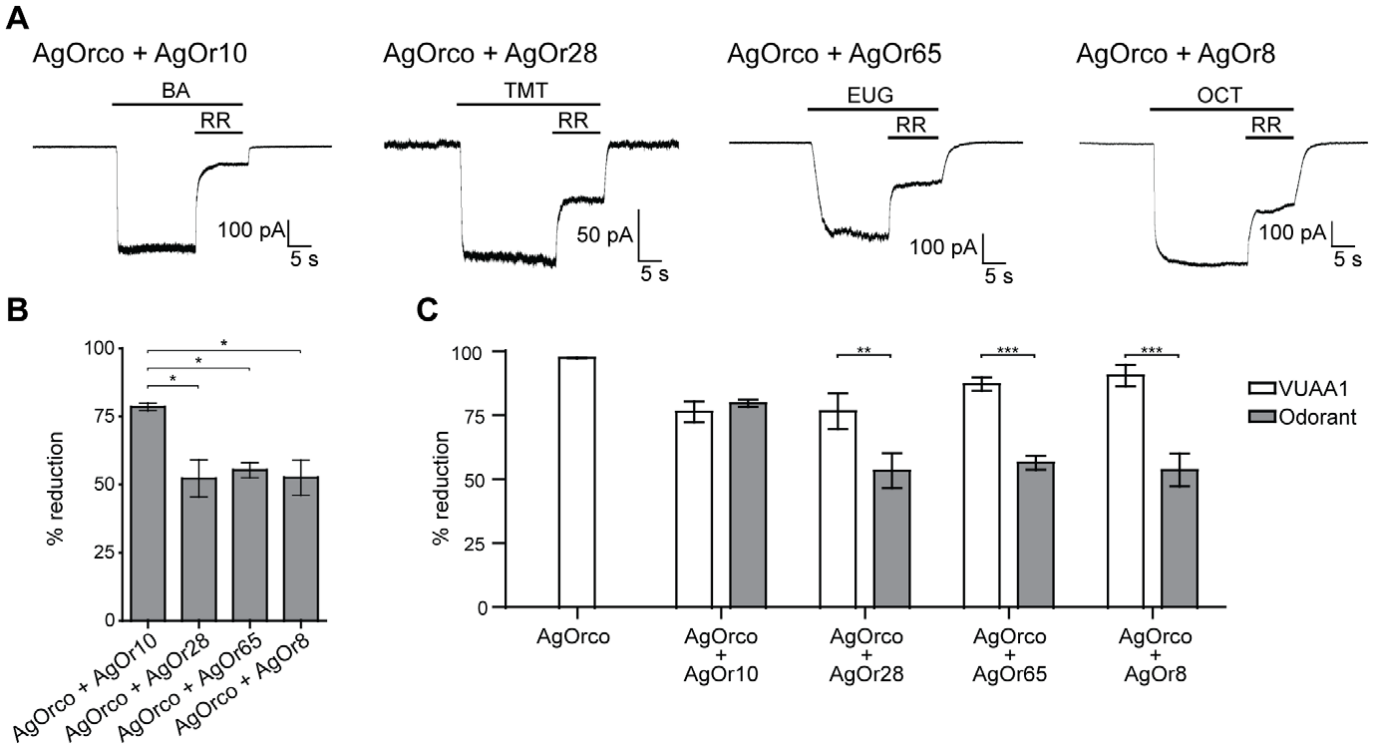
Susceptibility to RR depends on the AgOr and the agonist. (*A*) Representative traces of odorant-induced currents with subsequent current block by application of 100 µM RR. Odorant concentrations and abbreviations: 100 µM benzaldehyde (BA), 100 µM 2,4,5-trimethylthiazole (TMT), 100 nM eugenol (EUG), 100 µM 1-octen-3-ol (OCT). Holding potential for each recording is −60 mV. (*B*) The percent current reduction upon RR application across each AgOr combination (n = 5 for each). Statistical significance was determined by a one-factor ANOVA (p<0.01), and a Bonferroni correction was performed for individual comparisons (*  =  p<0.05). (*C*) Histogram comparing RR sensitivity by AgOr and agonist. Statistical significance was determined by a two-factor ANOVA (p<0.01), and a Bonferroni correction was performed for individual comparisons (***  =  p<0.001, **  =  p<0.01).

RR susceptibility was also examined when AgOr-expressing cells were stimulated by strong odorant agonists. A previous study on insect ORs found that odorants were also noncompetitive with RR blockade [7]. Here, AgOrco + AgOr10 currents were reduced by 78.5±1.4%, a significantly higher reduction than the other three AgOr complexes (Figure 6*A*–*B*). With the exception of AgOrco + AgOr10, each AgOrco + AgOr combination demonstrated significantly less reduction of odorant-induced currents when compared to VUAA1 agonism (Figure 6*C*). These results suggest that the odorant-specific AgOr influences the channel's susceptibility to RR and agree with previous results with *Drosophila* ORs, providing further support for its contribution to pore diversity among the OR ion channels in *An. gambiae* [7].

## Discussion

This study of the channel properties across a diverse set of AgOr complexes provides compelling evidence that the conventional OR, known to impart odorant specificity, also significantly contributes to the function of the channel pore. We observed that all of the AgOr complexes used in this study displayed an Eisenman III cation permeability sequence, and significant differences in the relative permeability of some individual ions were observed between conventional AgOrs coexpressed with AgOrco in the context of both VUAA1 and odorant-evoked responses. While the differences in permeability between the AgOrco + AgOr complexes in the VUAA1 studies could potentially be affected by a mixed population of AgOrco homomers, the overall variance between AgOrco-only cells and the AgOrco + AgOr cells indicates that the conventional AgOr can influence the cation permeability in the heteromeric channel.

Similarly, differences in RR sensitivity across the different AgOr complexes are consistent with the hypothesis that different heteromeric ORs have structurally distinct channel pores, in agreement with a previous study observing differences in RR susceptibility in a subset of *Drosophila* OR complexes [7]. Furthermore, while Rb^+^ was the most permeant cation among the AgOr complexes in this study, we note that a Rb^+^ gradient is not commonly established in biological systems. Interestingly, reports have found high concentrations of K^+^ (∼200 mM) in the sensillum lymph of moths [25], [26]. Together with the observed relative permeability of K^+^ in AgOr complexes, it is possible that influx of K^+^ may significantly contribute to depolarizing ORNs *in vivo*, in addition to Na^+^ and Ca^++^, which typically have favorable gradients for cation influx.

While this study has characterized OR complexes from *An. gambiae*, these data support a molecular model that should broadly apply to OR-mediated olfactory signaling across insects. Though these data cannot conclusively rule out the possibility of the conventional OR indirectly altering the channel pore architecture, our data supports the newly proposed model in which both the Orco coreceptor and the conventional OR directly contribute to the channel pore, similar to different channel subunits surrounding the pores of cyclic nucleotide gated channels and those of the nicotinic acetylcholine receptor superfamily [9], [11]. In this model, the conventional OR subunit that is responsible for odorant recognition has direct access to the channel pore where it can theoretically facilitate direct channel gating [1], [7]. Comparable to other ion channels, one subunit, Orco, can form functional homomeric channels in the absence of conventional OR [3], [9], [10]. The exact stoichiometry of Orco to the odorant-binding OR still remains as an important aspect in understanding the molecular mechanism of insect olfactory signaling.

The proposed model would have important implications for insect odor coding in that differences in odorant-evoked responses originate at the periphery, beginning with unique channel properties of each OR complex. The odorant-binding OR detects the specific odorant molecule, but it also can contribute to the qualitative and quantitative ability to flux cations through the OR channel pore. Along with the variables of OR expression, temporal dynamics of odorant mixtures, ORN morphology, and odorant concentration, the differences in the conductive properties of individual ORs may play a significant role in odorant-evoked depolarization of the ORN, which may ultimately result in propagation of the signal through an action potential [15], [27]. These findings define the additional role for conventional ORs in establishing the ion channel characteristics of insect ORs that goes significantly beyond odorant specificity.

## Materials and Methods

### Chemicals

VUAA1 (N-(4-ethylphenyl)-2-((4-ethyl-5-(3-pyridinyl)-4H-1,2,4-triazol-3-yl)thio)acetamide) was purchased from ChemBridge corporation (ID# 7116565). Benzaldehyde (CAS 100-52-7), 2,4,5-trimethylthiazole (CAS 13623-11-5), eugenol (CAS 97-53-0), 1-octen-3-ol (CAS 3391-86-4), and ruthenium red (CAS 11103-72-3) were all purchased from Sigma-Aldrich. All compounds were first dissolved in DMSO and subsequently diluted in external solution.

### Cell Culture, Ca^++^ Imaging, and Patch Clamp Electrophysiology

Generation of AgOrco + AgOrX cell lines and Ca^++^ imaging assays was performed as previously described [3], [19]. AgOr expression was induced by incubation with 0.3 µg/mL tetracycline for 18–42 hours before functional assays.

Whole-cell patch clamp recording from AgOr-expressing HEK cells were performed as previously described [3]. For cation permeability assays, the external solution for monovalent cations contained 150 mM *X*Cl, 1 mM MgCl_2_, 10 mM glucose, 10 mM HEPES, pH = 7.4 (*X* =  Li, Na, K, Rb, or Cs) [28]. The divalent cation external solution contained 30 mM *X*Cl_2_, 120 mM NMDG-Cl, 1 mM MgCl_2_, 10 mM glucose, 10 mM HEPES, pH 7.4 (*X* =  Ca or Mg). The internal (pipette) solution for cation permeability assays contained 150 mM NaCl, 1 mM MgCl_2_, 4 mM Na_2_ATP, 0.037 mM CaCl_2_, 5 mM EGTA, 10 mM HEPES, pH 7.2. The standard external solution for ruthenium red susceptibility assays contained 130 mM NaCl, 34 mM glucose, 10 mM HEPES, 1.5 mM CaCl_2_, 1.3 mM KH_2_PO_4_, and 0.5 mM MgSO_4_, pH 7.35 and the standard internal solution contained 120 mM KCl, 30 mM glucose, 10 mM HEPES, 2 mM MgCl_2_, 1.1 mM EGTA, and 0.1 mM CaCl_2_, pH 7.35.

To determine cation permeability, the agonist-induced current (−60 mV) was allowed to reach a steady state, and then a 2-second voltage ramp from −60 mV to +60 mV was applied to measure the reversal potential for each cation. Recordings were performed at room temperature (20–22°C) and reversal potentials were corrected for liquid junction potentials using pCLAMP 10 (Axon Instruments) under the Ag-AgCl wire reference electrode parameter (note that all current-voltage relationship traces in Figures (1–4) are not corrected for liquid junction potential). The ruthenium red protocol consisted of agonist application to steady-state current followed by the application of 100 µM ruthenium red with agonist. Percent current reduction was calculated from steady-state currents before and during ruthenium red application.

### Relative Permeability Calculations

The relative permeability of each monovalent cation to sodium was calculated according to the following equation: 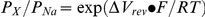
where Δ*V*_rev_ is the difference in reversal potential between the specific cation and sodium [28]. Permeability of divalent cations was calculated using the following equation:
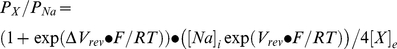
where *V*_rev_ is the absolute reversal potential of the divalent cation, [Na]*_i_* represents the intracellular sodium concentration, and [X]*_e_* is the extracellular concentration of the specific divalent cation [28]. Relative permeabilities can be found in Table S1.

Significant differences in cation permeability of different AgOr combinations were determined by ANOVA and post-hoc comparisons were made using a Bonferroni correction.

## Supporting Information

Figure S1 **Cells expressing only AgOrco do not respond to odorants.** The holding potential for each recording is −60 mV (n = 5). Concentrations and abbreviations: 100 µM benzaldehyde (BA), 100 µM 2,4,5-trimethylthiazole (TMT), 100 nM eugenol (EUG), 100 µM 1-octen-3-ol (OCT), 100 µM VUAA1.
(TIF)

Figure S2 **Comparison of monovalent cation permeability by agonist from** **Figures 1** **and** **2** **.** Odorant concentrations and abbreviations: 100 µM benzaldehyde (BA), 100 µM 2,4,5-trimethylthiazole (TMT), 100 nM eugenol (EUG), 100 µM 1-octen-3-ol (OCT). Statistical significance was determined by a two-factor ANOVA (p<0.05), and a Bonferroni correction was performed for individual comparisons (***  =  p<0.001, **  =  p<0.01, *  =  p<0.05).
(TIF)

Figure S3 **Comparison of divalent cation permeability by agonist from** **Figures 3** **and** **4** **.** Odorant concentrations and abbreviations: 100 µM benzaldehyde (BA), 100 µM 2,4,5-trimethylthiazole (TMT), 100 nM eugenol (EUG), 100 µM 1-octen-3-ol (OCT). Statistical significance was determined by a two-factor ANOVA (p<0.05), and a Bonferroni correction was performed for individual comparisons (**  =  p<0.01, *  =  p<0.05).
(TIF)

Table S1 **The relative permeabilities of the AgOrs to the mono- and divalent cations in the contexts of both VUAA1 and odorant agonism.**
(DOC)

Table S2 **Activation kinetics for responses to 100 µM VUAA1.** The 10–90% activation time was calculated using the statistics tool in pCLAMP 10 (Axon Instruments), and subsequent statistical significance was determined through a one-factor ANOVA and a post-hoc Bonferroni correction.
(DOC)
