# Supplementary figures and images for: Heteromeric Anopheline Odorant Receptors Exhibit Distinct Channel Properties
Source: PLoS One. 2011 Dec 9;6(12):e28774. doi: 10.1371/journal.pone.0028774 (PMC3235152; doi:10.1371/journal.pone.0028774)

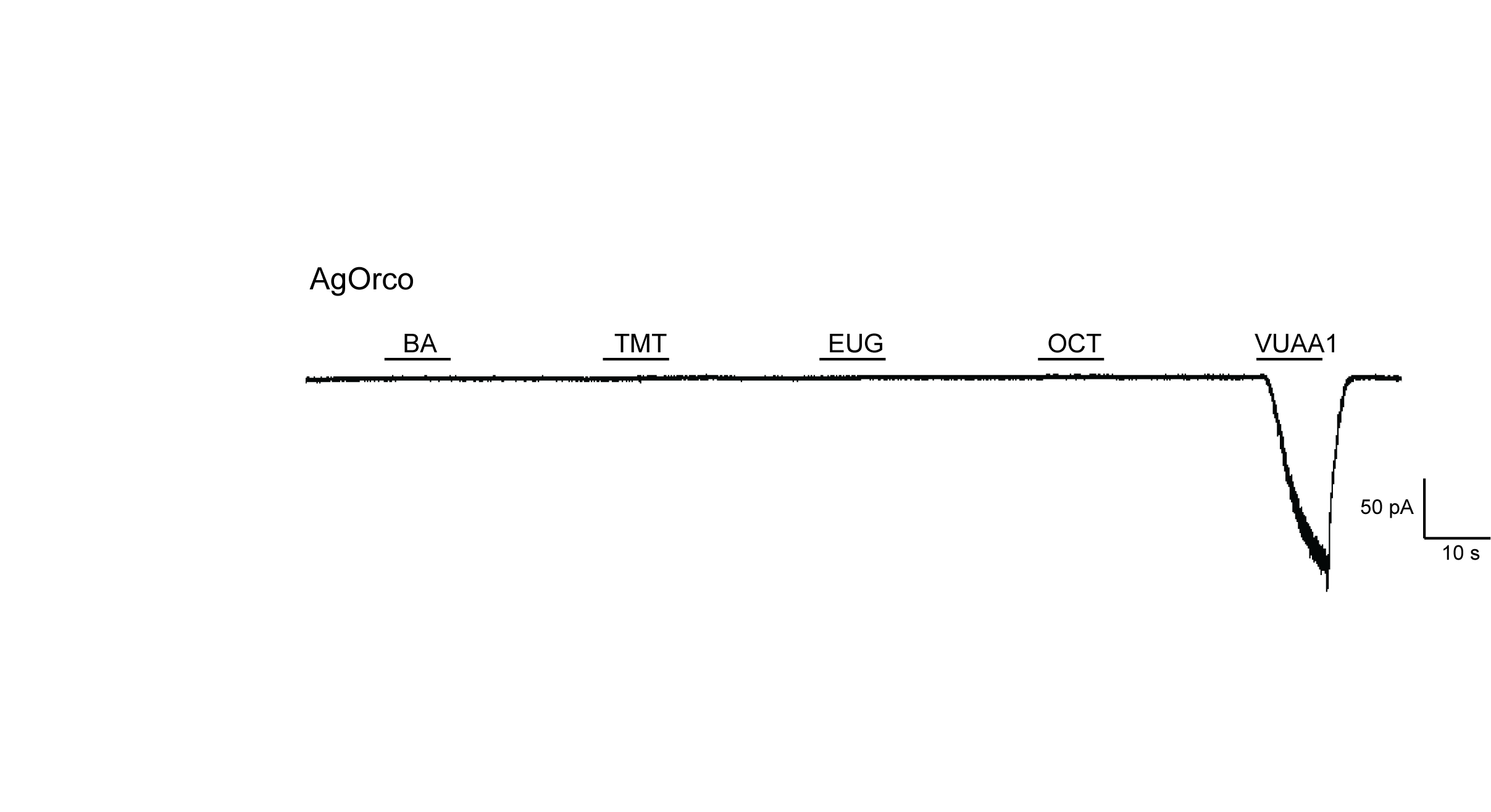

Supplement: Figure S1 — Cells expressing only AgOrco do not respond to odorants. The holding potential for each recording is −60 mV (n = 5). Concentrations and abbreviations: 100 µM benzaldehyde (BA), 100 µM 2,4,5-trimethylthiazole (TMT), 100 nM eugenol (EUG), 100 µM 1-octen-3-ol (OCT), 100 µM VUAA1. (TIF) [file pone.0028774.s001.tif]

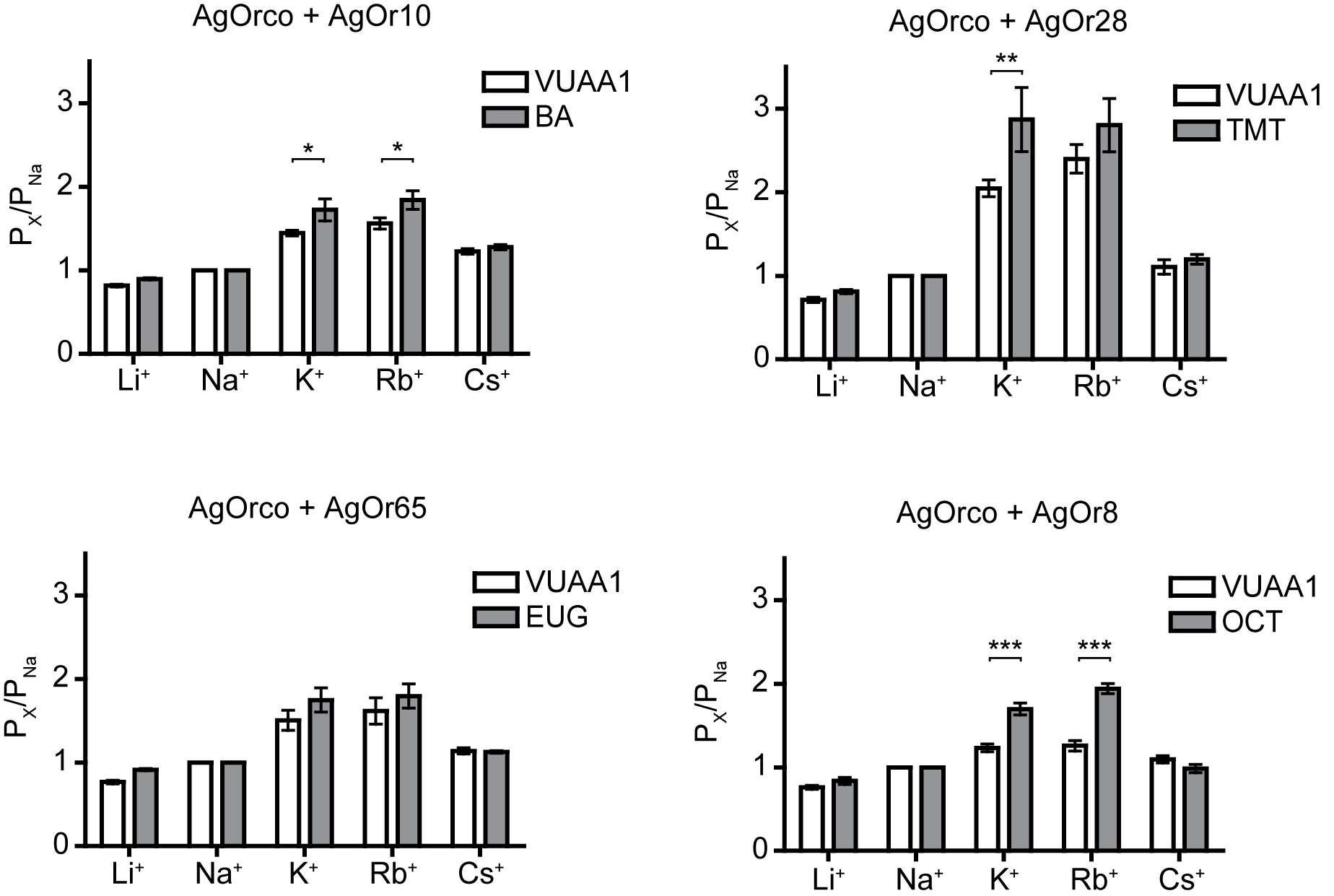

Supplement: Figure S2 — Comparison of monovalent cation permeability by agonist from Figures 1 and 2 . Odorant concentrations and abbreviations: 100 µM benzaldehyde (BA), 100 µM 2,4,5-trimethylthiazole (TMT), 100 nM eugenol (EUG), 100 µM 1-octen-3-ol (OCT). Statistical significance was determined by a two-factor ANOVA (p<0.05), and a Bonferroni correction was performed for individual comparisons (*** = p<0.001, ** = p<0.01, * = p<0.05). (TIF) [file pone.0028774.s002.tif]

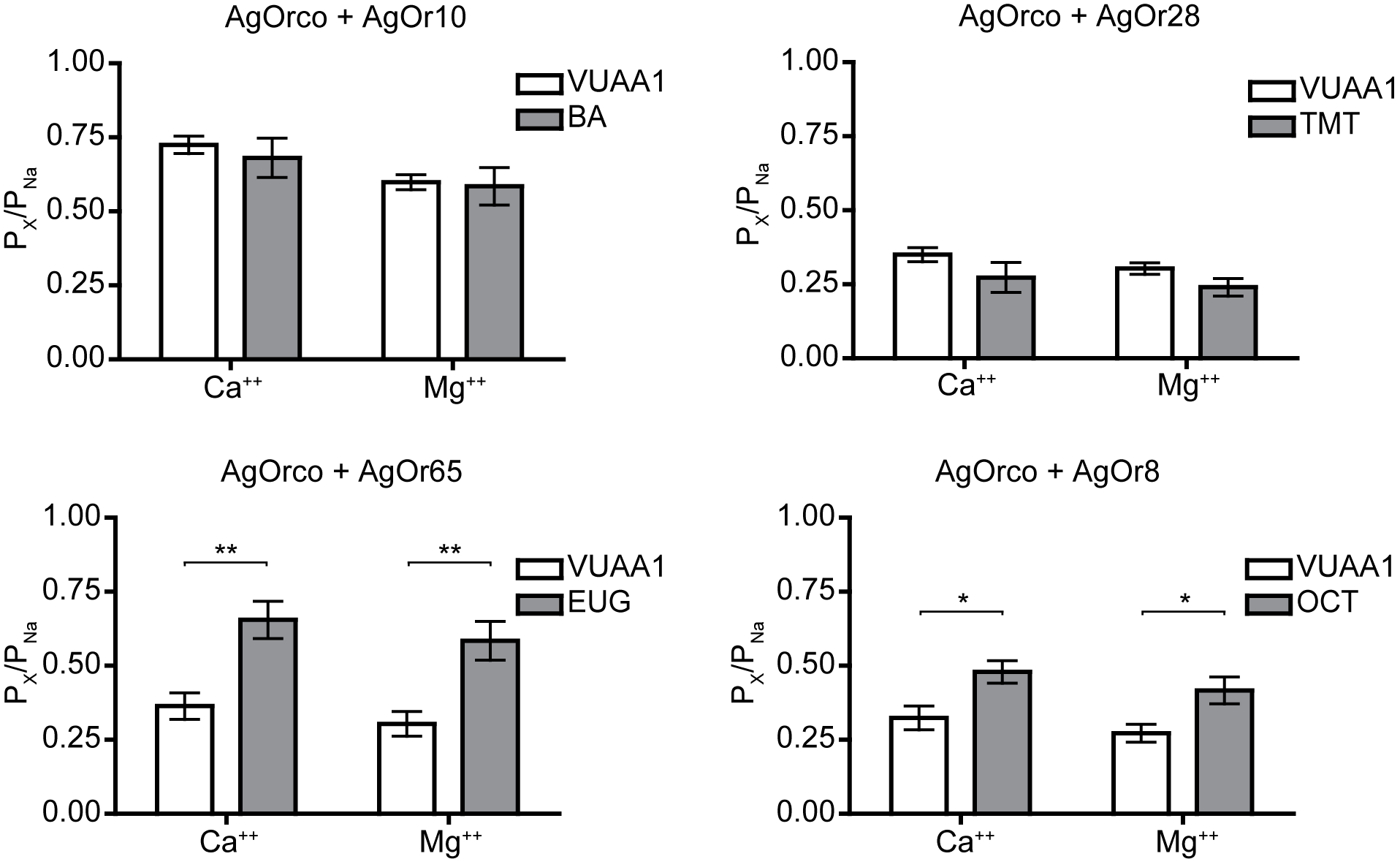

Supplement: Figure S3 — Comparison of divalent cation permeability by agonist from Figures 3 and 4 . Odorant concentrations and abbreviations: 100 µM benzaldehyde (BA), 100 µM 2,4,5-trimethylthiazole (TMT), 100 nM eugenol (EUG), 100 µM 1-octen-3-ol (OCT). Statistical significance was determined by a two-factor ANOVA (p<0.05), and a Bonferroni correction was performed for individual comparisons (** = p<0.01, * = p<0.05). (TIF) [file pone.0028774.s003.tif]
