# Supplementary material for: Heteromeric Anopheline Odorant Receptors Exhibit Distinct Channel Properties
Source: PLoS One. 2011 Dec 9;6(12):e28774. doi: 10.1371/journal.pone.0028774 (PMC3235152; doi:10.1371/journal.pone.0028774)
Supplement: Table S1 — The relative permeabilities of the AgOrs to the mono- and divalent cations in the contexts of both VUAA1 and odorant agonism. (DOC) [file pone.0028774.s004.doc]

| **AgOr** | **Agonist** | ***P*_Rb_/*P*_Na_** | ***P*_K_/*P*_Na_** | ***P*_Cs_/*P*_Na_** | ***P*_Li_/*P*_Na_** | ***P*_Ca_/*P*_Na_** | ***P*_Mg_/*P*_Na_** |
| --- | --- | --- | --- | --- | --- | --- | --- |
| AgOrco | VUAA1 | 1.82 ± 0.08 | 1.60 ± 0.05 | 1.19 ± 0.04 | 0.73 ± 0.02 | 0.34 ± 0.03 | 0.31 ± 0.02 |
| AgOrco + AgOr10 | VUAA1 | 1.56 ± 0.07 | 1.45 ± 0.03 | 1.22 ± 0.03 | 0.81 ± 0.02 | 0.72 ± 0.03 | 0.60 ± 0.03 |
|  | benzaldehyde | 1.84 ± 0.11 | 1.72 ± 0.13 | 1.28 ± 0.03 | 0.90 ± 0.01 | 0.68 ± 0.07 | 0.58 ± 0.06 |
| AgOrco + AgOr28 | VUAA1 | 2.40 ± 0.17 | 2.05 ± 0.10 | 1.11 ± 0.09 | 0.71 ± 0.03 | 0.35 ± 0.02 | 0.30 ± 0.02 |
|  | 2,4,5-tri-methylthiazole | 2.80 ± 0.32 | 2.87 ± 0.38 | 1.19 ± 0.06 | 0.81 ± 0.02 | 0.27 ± 0.05 | 0.24 ± 0.03 |
| AgOrco + AgOr65 | VUAA1 | 1.62 ± 0.16 | 1.51 ± 0.12 | 1.14 ± 0.04 | 0.77 ± 0.02 | 0.36 ± 0.05 | 0.30 ± 0.04 |
|  | eugenol | 1.80 ± 0.15 | 1.75 ± 0.14 | 1.13 ± 0.01 | 0.92 ± 0.01 | 0.66 ± 0.06 | 0.58 ± 0.07 |
| AgOrco + AgOr8 | VUAA1 | 1.25 ± 0.06 | 1.23 ± 0.05 | 1.10 ± 0.04 | 0.76 ± 0.02 | 0.32 ± 0.04 | 0.27 ± 0.03 |
|  | 1-octen-3-ol | 1.94 ± 0.06 | 1.70 ± 0.07 | 0.98 ±0.04 | 0.84 ± 0.04 | 0.48 ± 0.04 | 0.42 ± 0.05 |

**Table S1. The relative permeabilities of the AgOrs to the mono- and divalent cations in the contexts of both VUAA1 and odorant agonism**
