# Supplementary material for: Heteromeric Anopheline Odorant Receptors Exhibit Distinct Channel Properties
Source: PLoS One. 2011 Dec 9;6(12):e28774. doi: 10.1371/journal.pone.0028774 (PMC3235152; doi:10.1371/journal.pone.0028774)
Supplement: Table S2 — Activation kinetics for responses to 100 µM VUAA1. The 10–90% activation time was calculated using the statistics tool in pCLAMP 10 (Axon Instruments), and subsequent statistical significance was determined through a one-factor ANOVA and a post-hoc Bonferroni correction. (DOC) [file pone.0028774.s005.doc]

| **AgOr** | **10-90% activation time (sec)** |
| --- | --- |
| AgOrco | 13.6 ± 0.65 |
| AgOrco + AgOr10 | 5.53 ± 0.89^a^ |
| AgOrco + AgOr28 | 15.89 ± 0.60 |
| AgOrco + AgOr65 | 15.73 ± 0.27 |
| AgOrco + AgOr8 | 12.78 ± 1.40 |

^a^ Significantly different from each other receptor combination (p < 0.001)

**Table S2.** **Activation kinetics for responses to 100 μM VUAA1.** The 10-90% activation time was calculated using the statistics tool in pCLAMP 10 (Axon Instruments), and subsequent statistical significance was determined through a one-factor ANOVA and a post-hoc Bonferroni correction.
